# Global Profiling of 2-hydroxyisobutyrylome in Common Wheat

**DOI:** 10.1016/j.gpb.2020.06.008

**Published:** 2021-02-11

**Authors:** Ning Zhang, Lingran Zhang, Linjie Li, Junyou Geng, Lei Zhao, Yan Ren, Zhongdong Dong, Feng Chen

**Affiliations:** National Key Laboratory of Wheat and Maize Crop Science / Agronomy College, Henan Agricultural University, Zhengzhou 450046, China

**Keywords:** Post-translational modification, Lysine 2-hydroxyisobutyrylation, Common wheat, Proteomics, Co-immunoprecipitation

## Abstract

As a novel **post-translational modification** (PTM), **lysine 2-hydroxyisobutyrylation** (Khib) is considered to regulate gene transcriptional activities in eukaryotic cells; however, the functions of Khib-modified proteins in plants remain unknown. Here, we report that Khib is an evolutionarily-conserved PTM in wheat and its progenitors. A total of 3348 Khib sites on 1074 proteins are identified in **common wheat** (*Triticum aestivum* L.) by using affinity purification and mass spectroscopy of 2-hydroxyisobutyrylome. Bioinformatic data indicate that Khib-modified proteins participate in a wide variety of biological and metabolic pathways. Immunoprecipitation confirms that Khib-modified proteins are present endogenously. A comparison of Khib and other main PTMs shows that Khib-modified proteins are simultaneously modified by multiple PTMs. Using mutagenesis experiments and **co-immunoprecipitation** assays, we demonstrate that Khib on K206 of phosphoglycerate kinase (PGK) is a key regulatory modification for its enzymatic activity, and mutation on K206 affects the interactions of PGK with its substrates. Furthermore, Khib modification of low-molecular-weight proteins is a response to the deacetylase inhibitors nicotinamide and trichostatin. This study provides evidence to promote our current understanding of Khib in wheat plants, including the cooperation between Khib and its metabolic regulation.

## Introduction

Protein post-translational modifications (PTMs) can change the charge, conformation, and molecular weight of proteins by adding chemical groups to the amino acid residues of a protein, which can also expand the biological functions of the protein [1]. PTMs have been found to play a vital role in diverse biological processes by regulating protein functions [2]. Lysine acetylation (Kac) is an important PTM that neutralizes positively-charged lysine residues; previous studies of Kac have mainly focused on nuclear proteins [3], [4]. Using mass spectrometry, a high abundance of non-histone proteins has been extensively characterized recently [5], [6], [7], [8], [9]. Kac is currently known to regulate diverse protein properties, including subcellular localization, DNA–protein interactions, protein stability, protein–protein interactions (PPIs), and enzymatic activity [7], [8], [10], [11]. With the help of high-sensitivity mass spectrometry, there are currently nine novel lysine PTMs reported, including formylation, crotonylation, butyrylation, succinylation (Ksu), malonylation (Kma), propionylation, glutarylation, β-hydroxybutyrylation, and 2-hydroxyisobutyrylation (Khib), and the catalog is still growing [12], [13], [14], [15], [16], [17], [18]. These novel PTMs have been mainly found in mammalian and yeast cells, and also in plants, such as *Arabidopsis thaliana* [19], rice [20], [21], [22], [23], and tobacco [19], [24]. Increasing evidence suggests that these new types of PTMs are involved in multiple kinds of cellular and metabolic pathways in plants [20].

The wheat genome is large, and its advancements are slower compared with other plants; however, some progress has been made. To date, only two novel PTMs, Ksu and Kma, have been identified in wheat [25], [26], [27]. These new acylation types were initially identified in the ε-amino group on the lysines of core histones [25], [26], while recent studies have indicated that nuclear, cytoplasmic, and mitochondrial proteins can also harbor lysine acylation [28]; this is because all acylation types require acyl-CoAs as corresponding donors. In addition, lysine PTMs have been found to participate in diverse cellular metabolic pathways [2], [8], [29], [30], [31], [32], [33], [34]. Altogether, these studies have dramatically enriched our understanding of histone modifications. As a next step, many researchers have started investigating the potential function of histone modifications in transcription as well as the enzymes involved in adding (writers), removing (erasers), and reading (readers) PTMs.

As a new type of histone marker, Khib has been found to be conserved in the genomes of many species including yeast and human. At present, Khib has been initially identified in 63 lysine sites on human and mouse histones and could play a critical role in the regulation of chromatin functions [16]. Khib is also important for histone 4 lysine 8 (H4K8), as H4K8hib is associated with gene transcription in meiotic and post-meiotic cells [16]. Moreover, a recent study has indicated that Khib on H4K8 is regulated by glucose homeostasis in *Saccharomyces cerevisiae*, where histone lysine deacetylases Rpd3p and Hos3p function as regulatory enzymes for lysine de-Khib reactions for H4K8 [35]. Eliminating Khib on H4K8 resulted in a reduced life span, implying that the modification of the H4K8 site may be involved in aging [35].

Over the past several years, many regulatory enzymes (writers and erasers) have been identified [36]. A study has indicated that histone deacetylase 2 (HDAC2) and HDAC3 act as “erasers” in mammalian cells, and Esa1p and its homologue Tip60 serve as “writers” in budding yeast and human cells, respectively [37]. Another study has reported that the histone lysine acetyltransferase EP300 is a ‘‘writer’’ for Khib in mammalian cells, and it is able to regulate glycolysis through Khib of glycolytic enzymes and mediate cell survival via nutritional regulation through glycolysis [38]. A very recent study has indicated that CobB serves as a de-Khib enzyme that regulates glycolysis and cell growth in bacteria [39]. Proteome-wide profiling of Khib in mammalian cells, yeast, *Proteus mirabilis*, rice, and *Physcomitrella patens* has discovered several nuclear, cytosolic, and mitochondrial proteins with acetyllysine modifications, providing evidence that Khib may play a vital role in cell metabolism; this has stimulated research on the non-nuclear functions of Khib [23], [35], [37], [40], [41]. Progress is made mainly in mammalian cells and yeast; studies in plants are still limited. Therefore, it is interesting to determine the functions of Khib-modified proteins in plants.

In this study, we analyzed wheat Khib sites by utilizing high performance liquid chromatography (HPLC) and tandem mass spectroscopy (MS/MS). A total of 3348 Khib sites on 1074 proteins were identified in common wheat (*Triticum aestivum* L.). Using mutagenesis experiments, we confirmed that Khib on K206 of phosphoglycerate kinase (PGK) was a key regulatory modification; we also observed that the interactions of PGK with its substrates were impacted by Khib. Western blotting (WB) results indicated that wheat sirtuins (SIRTs) or Zn-binding HDACs regulated Khib of low-molecular-weight proteins. Our data indicated that the broad regulatory scope of Khib was comparable with that of other major PTMs. These novel Khib sites and proteins not only expand our understanding of the functional roles of Khib but also lay a foundation for future studies in plants.

## Results and discussion

### Evolutionary conservation of Khib

**Figure 1:**
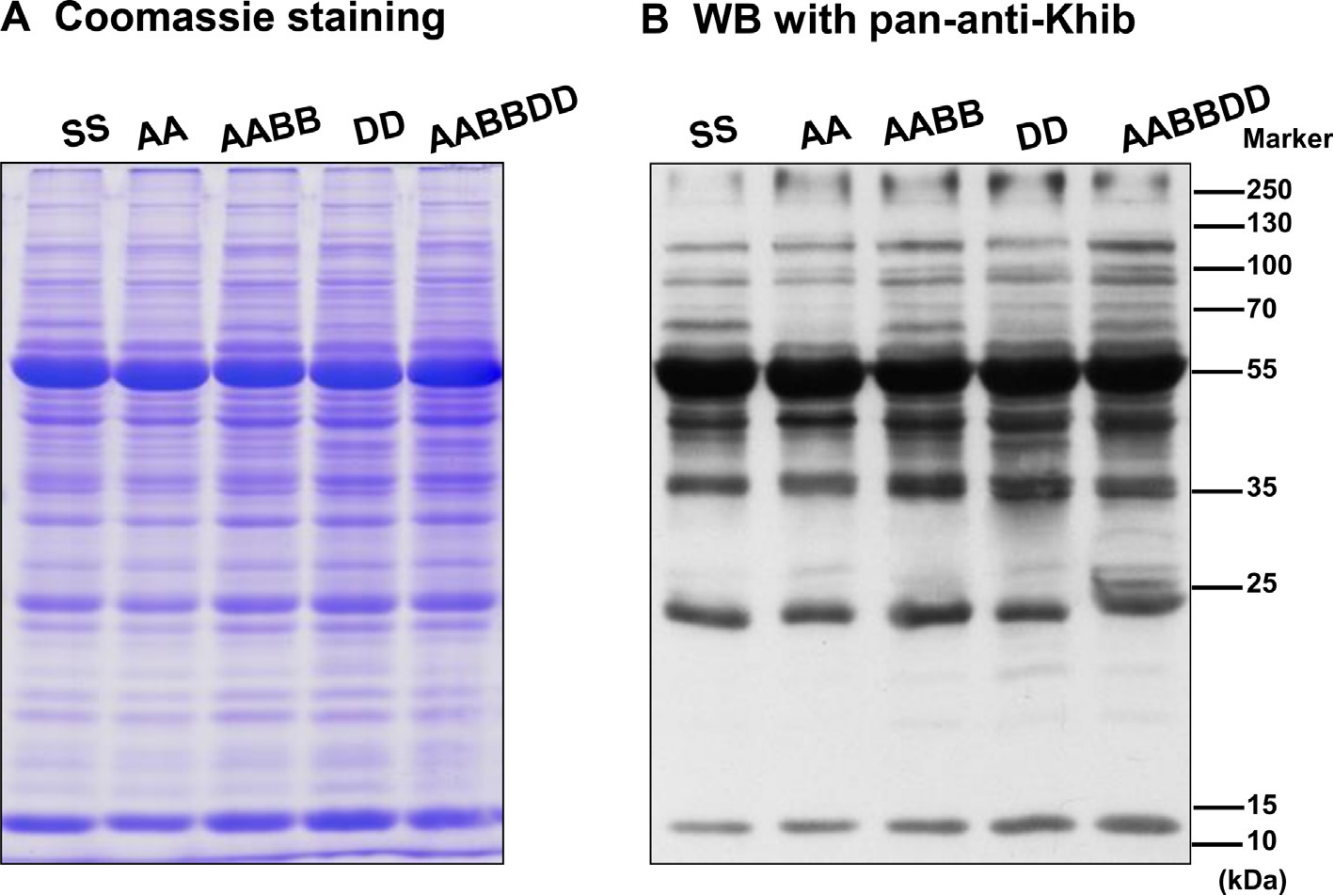
**Khib is an evolutionarily-conserved PTM** **A.** SDS-PAGE gel. **B.** WB of the Khib-modified proteins in wheat and its progenitors. SS, *Aegilops speltoides*; AA, *Triticum Urartu*; AABB, *Triticum durum*; DD, *Aegilops tauschii*; AABBDD, *Triticum aestivum* L.; Khib, lysine 2-hydroxyisobutyrylation; PTM, post-translational modification; SDS-PAGE, sodium dodecyl sulfate-polyacrylamide gel electrophoresis; WB, Western blotting; pan-anti-Khib, pan-anti-2-hydroxyisobutyryllysine antibody.

Previous studies have indicated that histone Khib is an evolutionarily-conserved PTM in eukaryotic cells (*e.g.*, HeLa cells, *Drosophila* S2, mouse embryonic fibroblasts, and yeast *S. cerevisiae* cells) [16]. To test whether Khib is present in wheat and its progenitors, the pan-anti-2-hydroxyisobu-tyryllysine (pan-anti-Khib) antibody was used for WB analyses in *Triticum urartu* (PI428185, AA), *Aegilops speltoides* (PI542245, SS), *Aegilops tauschii* var. *strangulate* (AS2393, DD), tetraploid *Triticum durum* Langdon (AABB), and hexaploid wheat Aikang 58 (Ak58, AABBDD) (Figure 1). Our analyses revealed that Khib was widely distributed and mainly enriched in 40–70 kDa in hexaploid wheat and its progenitors. Hexaploid wheat had the highest Khib level compared with its progenitors. Additionally, the Khib levels of the diploid progenitors (AA, SS, and DD) were lower than that of the tetraploid progenitor (AABB). These results indicate that Khib is an evolutionarily-conserved PTM in wheat and its progenitors.

### Global analysis of wheat 2-hydroxyisobutyrylome

#### The lysine 2-hydroxyisobutyrylome profile

**Figure 2:**
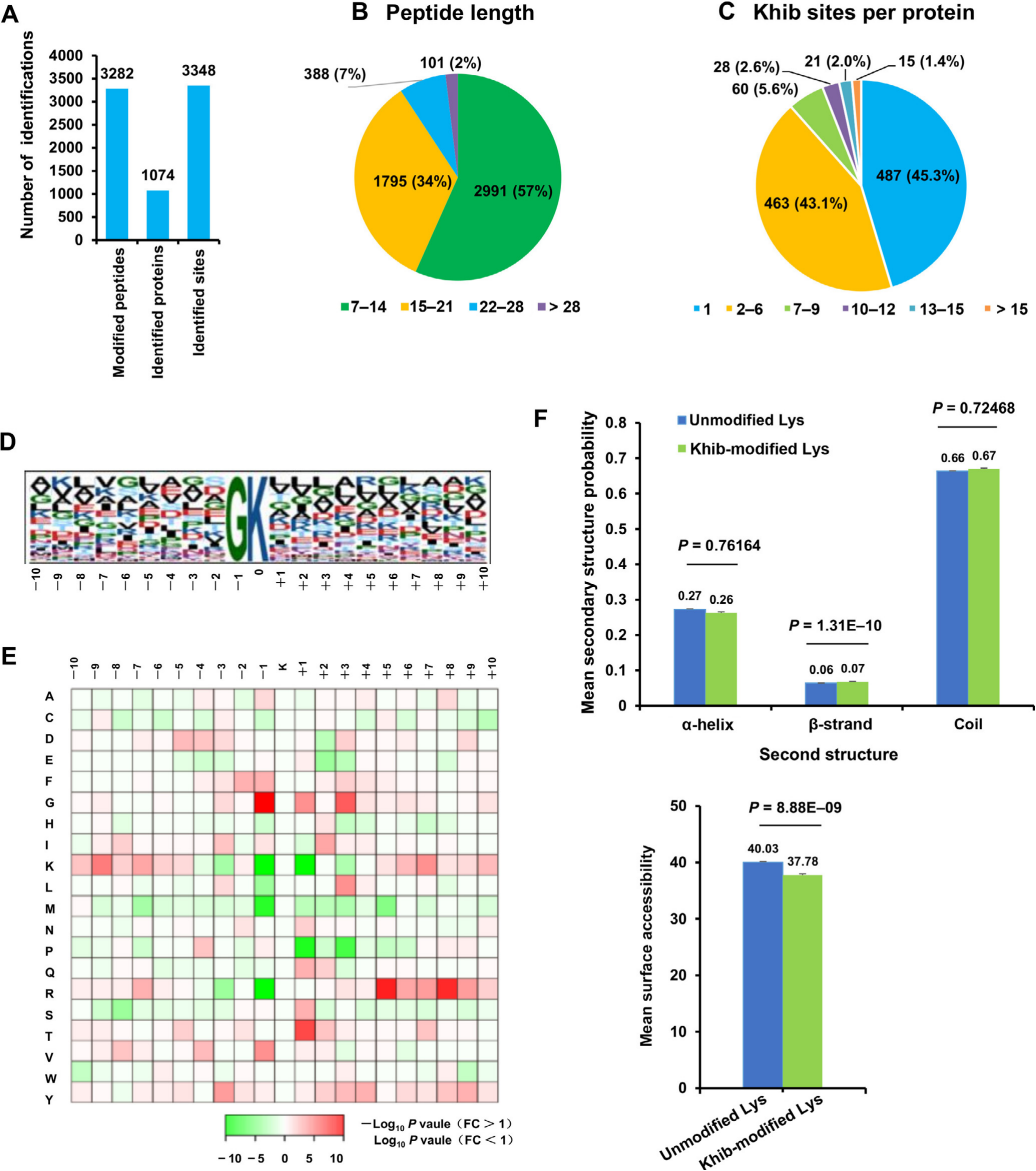
**Proteome-wide identification and properties of Khib sites in wheat** **A.** Bar chart showing the numbers of Khib sites, modified proteins, and modified peptides. **B.** Peptide length distribution. **C.** The distribution of proteins with different numbers of Khib sites. **D.** Khib motifs and the conservation of Khib sites. The height of each letter represents its frequency, and the central K represents the Khib-modified lysine. **E.** Heatmap of amino acid compositions of the Khib sites. **F.** Probabilities of Khib in three different protein secondary structures (upper) and the predicted surface accessibility of Khib-modified lysine residues (lower). Lys, lysine; FC, fold change.

**Supplementary Figure S2:**
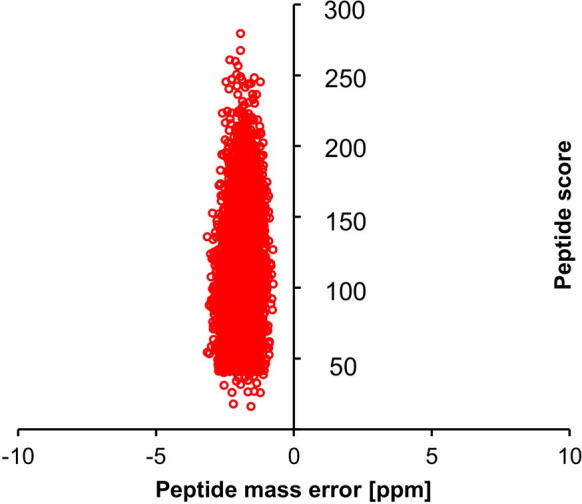
**Mass error distribution of identified peptides in common wheat**

A proteome analysis was conducted using the antibody-based affinity enrichment and LC-MS/MS methods. A total of 3348 Khib sites on 1074 proteins in hexaploid wheat were identified (Figure 2A, Figure S1; Table S1). Most of the mass errors were less than 5 ppm, confirming the high accuracy of our MS data (Figure S2). The length of most peptides (98%) was in line with the properties of tryptic peptides (from 7 to 28 amino acids) (Figure 2B). To evaluate the coverage of Khib in substrate proteins, we counted the number of Khib sites per protein. Of the 1074 Khib-modified proteins, 45.3% and 43.1% have 1 and 2–6 Khib sites, respectively, while the remaining 11.6% have ≥ 7 Khib sites (Figure 2C), indicating that 54.7% contained multiple Khib sites. Wheat 2-hydroxyisobutyrylome was larger than what has been previously reported for acetylome, malonylome, succinylome, and ubiquitome in plants [42], [25], [26], [27]. This is in agreement with a previous study, which has indicated that there are more Khib sites on histones than other PTM sites [16], implying that Khib is an abundant PTM and may play a vital role in substrate protein regulation.

Sequence motifs in Khib-modified peptides were determined by the flanking sequences of these sites using the Motif-X program. According to the heatmap of amino acid compositions at the Khib sites, considerable non-polar hydrophobic amino acid glycine (G) was enriched at the −1 position, indicating that there is likely Khib at this location in wheat (Figure 2D and E). This pattern was different from Khib motifs in rice, *Proteus mirabilis*, and humans [23], [39], [40], although GKhib simultaneously exists in *P. patens* proteins [41]. This surprising result indicates the complexity of underlying PTM mechanisms in plants.

We conducted a structural analysis of all identified proteins using NetSurfP. Results indicated that 26% and 7% of the Khib sites were located in α-helices and β-strands, respectively, while 67% of the Khib sites were located in disordered coils (Figure 2F; Table S2). Khib sites were found more frequently in β-strands (*P* = 1.31E−10) and less frequently in α-helices (*P* = 0.76164) and disordered coils (*P* = 0.72468) when compared with unmodified lysine residues. Thus, it is clear that Khib has a preference for secondary structures. Identified Khib sites were further evaluated for solvent accessibility, and it was found that 37.78% of Khib-modified lysine residues were exposed to the protein surface, compared with 40.03% of unmodified lysine residues (*P* = 8.88E−09) (Figure 2F; Table S2), suggesting that Khib-modified lysine residues are less surface-accessible compared to the unmodified ones. The lower surface accessibility of Khib-modified lysine residues implies that lysine Khib may occur in a selective process.

#### Enrichment analyses of the Khib-modified proteins

**Supplementary Figure S3:**
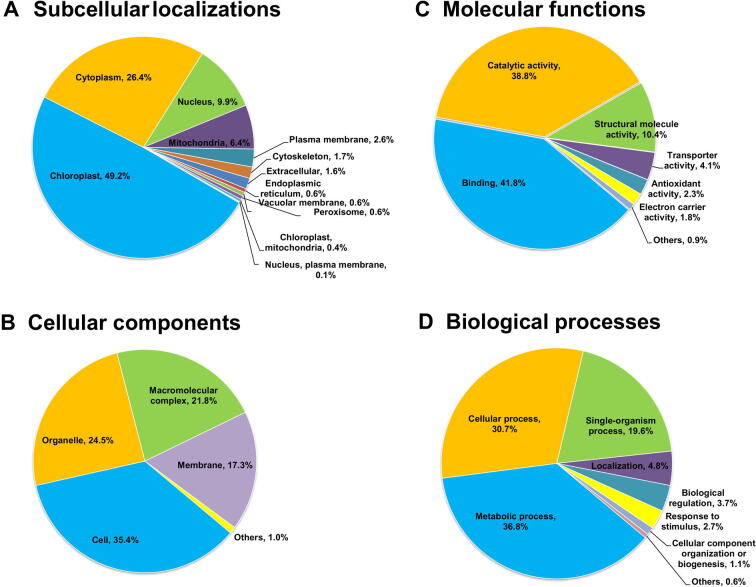
**Pie charts of the distribution of 2-hydroxyisobutyrylation proteins based on their predicted subcellular localizations (A), cellular components (B), molecular functions (C), and biological processes (D)**

To evaluate Khib-modified proteins, we performed the Gene Ontology (GO) functional classification according to subcellular location, cellular component, molecular function, and biological process (Figure S3A–C; Tables S3 and S4,). Based on subcellular location, there were 49.2%, 26.4%, 9.9%, and 6.4% Khib-modified proteins in the chloroplast, cytoplasm, nucleus, and mitochondria, respectively, indicating a wide distribution of Khib-modified proteins in wheat. Based on cellular component, Khib-modified proteins were distributed in cells (35.4%), organelles (24.5%), macromolecular complexes (21.8%), and membranes (17.3%). Based on molecular function analysis, 41.8% and 38.8% of Khib-modified proteins were associated with binding and catalytic activities, respectively; this could imply that Khib is an important PTM in DNA transcription or PPIs and has a large influence on metabolic processes. Biological process analysis showed that 36.8%, 30.7%, 19.6%, and 4.8% of Khib-modified proteins were involved in metabolism, cellular processes, single organism proteins, and location, respectively. These features are similar to the previous reports in rice and *P. patens*.

**Figure 3:**
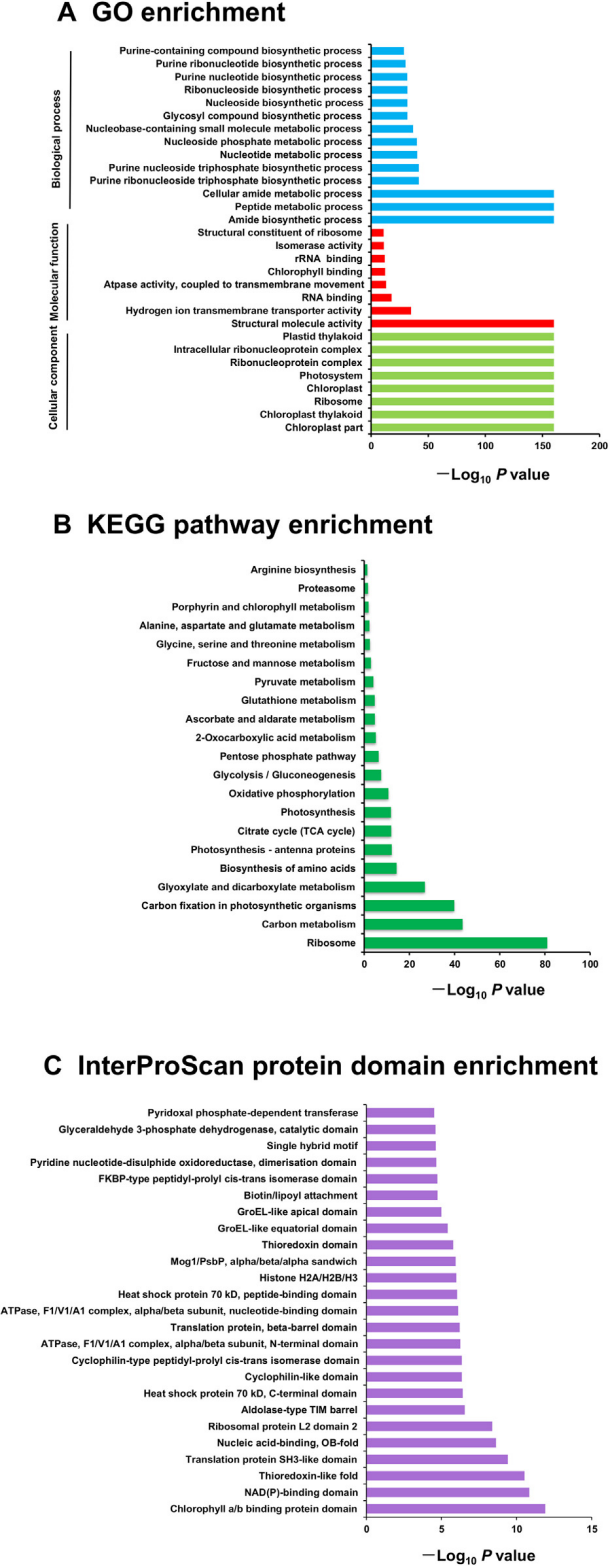
**Enrichment analyses of Khib-modified proteins in wheat** **A.** GO enrichment analysis. **B.** KEGG pathway enrichment analysis. **C.** Protein domain enrichment analysis. GO, Gene Ontology; KEGG, Kyoto Encyclopedia of Genes and Genomes.

To further understand the characteristics and potential roles of wheat Khib-modified proteins, we performed enrichment analyses of GO terms, Kyoto Encyclopedia of Genes and Genomes (KEGG) pathways, and protein domains (Figure 3A–C; Tables S5–S8). Biological process enrichment indicated that Khib-modified proteins were enriched in a variety of cellular and metabolic processes, indicating a wide impact of this novel PTM in wheat. Similar to Kac, Khib of metabolically related enzymes could have a conserved regulation pattern in different organisms [23], [35], [39], [40], [41]. KEGG enrichment analysis demonstrated that Khib-modified proteins were enriched in multiple primary metabolic pathways (Figure 3B), such as carbon metabolism, carbon fixation in photosynthetic organisms, and photosynthesis-antenna proteins. In contrast, ribosomes, biosynthesis of amino acids, proteasomes, and arginine biosynthesis were also significantly enriched in the KEGG pathway analysis, suggesting the potential role of Khib in the regulation of protein biosynthesis and degradation. Protein domain enrichment analysis showed that Khib-modified proteins were enriched in the chlorophyll a/b binding protein domain, NAD(P)-binding domain, thioredoxin-like fold, heat shock protein 70 kD, histone H2A/H2B/H3, and pyridoxal phosphate-dependent transferase (Figure 3C). Enrichment analysis of KEGG pathways using the InterPro Domain protein database indicated that photosynthesis and carbon metabolism are likely significantly regulated by Khib.

#### PPI prediction

**Supplementary Figure S4:**
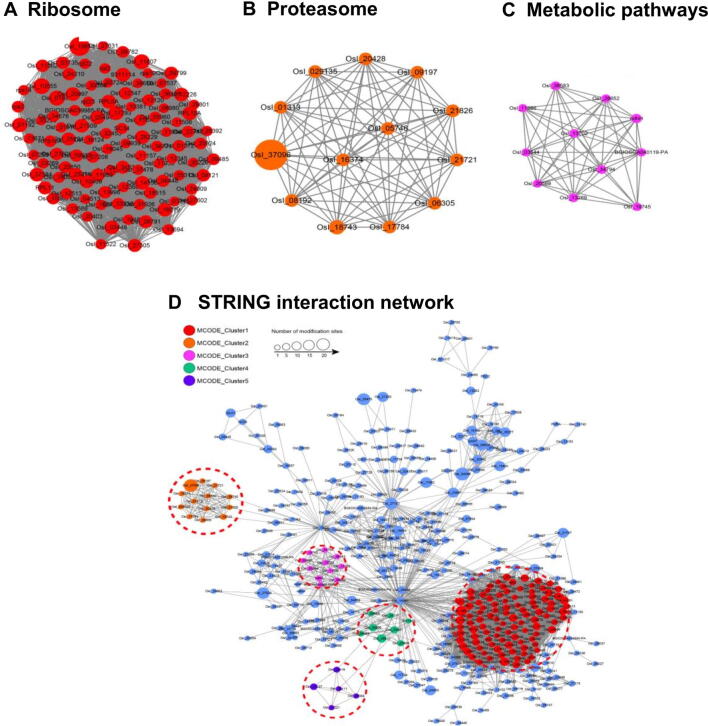
**Interaction networks of the 2-hydroxyisobutyrylation proteins in wheat using STRING A.** 102 ribosome-related proteins were mapped the interaction. **B.** 11 proteins associated with proteasome were mapped the interaction. **C.** 10 proteins involved in metabolic were mapped the interaction. **D.** 435 proteins were mapped the interaction.

To quantify PPI properties of the 2-hydroxyisobutyrylome, physical and functional interaction analysis was performed using STRING. We found the existence of connections among 435 Khib-modified proteins, indicating that Khib-modified proteins are able to participate in a diverse set of functions in wheat (Table S9; Figure S4A–D). We also retrieved 21 highly interconnected clusters of Khib-modified proteins using the Cytoscape software algorithm. For example, 102 ribosome-related proteins (Cluster 1), 11 proteasome-related proteins (Cluster 2), and 10 proteins associated with metabolic pathways (Cluster 3) demonstrated the presence of close interaction networks (Figure S4A–C). The pathway subnetworks suggested that all of 435 Khib-modified proteins participate in a dense PPI network.

#### Validation of Khib

**Figure 4:**
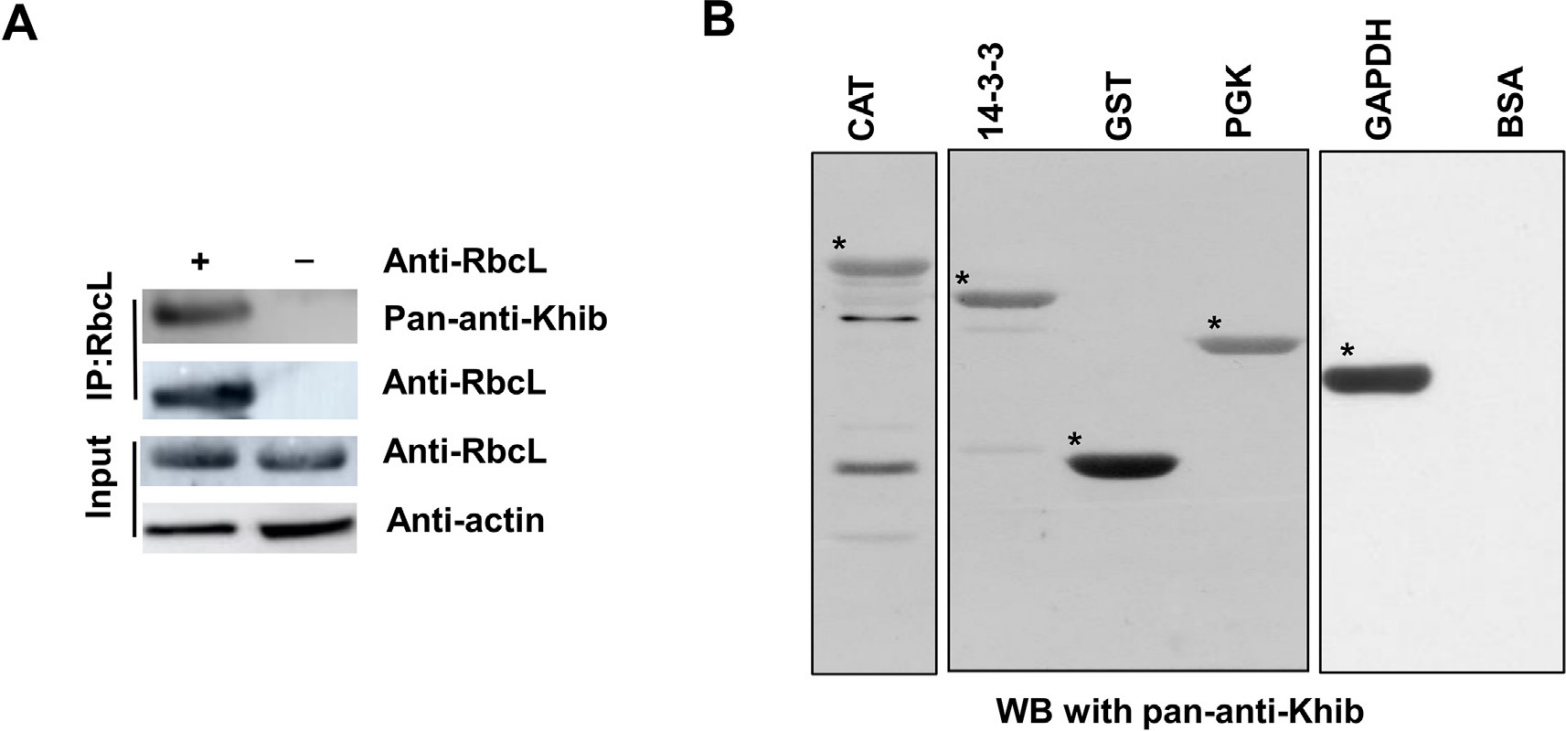
**Independent validation of Khib of proteins** **A.** IP of RbcL was performed with (+) or without (–) anti-RbcL antibodies; eluted proteins were probed with either pan-anti-Khib or anti-RbcL antibodies. **B.** Purified recombinant proteins and the positive control BSA were measured by WB. IP, immunoprecipitation; RbcL, the large subunit of ribulose-1,5-bisphosphate carboxylase; CAT, catalase; GST, glutathione S-transferase; GAPDH, glyceraldehyde-3-phosphate dehydrogenase; PGK, phosphoglycerate kinase; BSA, bovine serum albumin.

Six proteins were identified, and their Khib states were independently confirmed by immunostaining (Figure 4A and B; Table 1, Table S1). The large subunit of ribulose-1,5-bisphosphate carboxylase (RbcL) from wheat leaf proteins was immunoprecipitated by an anti-RbcL antibody, where Khib was clearly present (Figure 4A). Five proteins, *i.e.*, catalase (CAT), 14-3-3, glutathione S-transferase (GST), PGK, and glyceraldehyde-3-phosphate dehydrogenase (GAPDH) from the wheat germ protein expression system (cell free) were expressed as fusions with the His-tag and/or the Halo-tag and showed clear Khib signals; the positive control bovine serum albumin (BSA) did not have a Khib signal (Figure 4B). Together, these results demonstrate that 2-hydroxyisobutyrylome is present endogenously.

**Table 1:** Khib sites of proteins used for Co-IP and wheat germ protein expression system

**Protein name**	**Protein accession No.**	**Khib position**
RbcL	P11383	K7, K14, K18, K21, K32, K99, K146, K164, K175, K177, K183, K201, K227, K236, K252, K305, K316, K334, K356, K450
CAT	A0A1D5YMQ8	K50, K72, K125, K163, K223, K227, K401, K424, K429, K464, K481
14-3-3 protein	L0GED8	K36, K56, K75, K89, K101, K124, K129, K142, K148, K250
PGK	W5H4V7	K35, K58, K78, K137, K202, K206, K366
GAPDH	A0A1D5TTT4	K202, K206, K366, K167, K217, K222, K274, K276, K300, K301
GST	Q9SP56	K138, K140, K152

*Note*: Protein accession Nos. are from the UniProt database. K, lysine; Khib, lysine 2-hydroxyisobutyrylation; Co-IP, co-immunoprecipitation; RbcL, the large subunit of ribulose-1,5-bisphosphate carboxylase; CAT, catalase; GST, glutathione S-transferase; GAPDH, glyceraldehyde-3-phosphate dehydrogenase; PGK, phosphoglycerate kinase.

#### Conservation and uniqueness of the Khib-modified proteins

**Figure 5:**
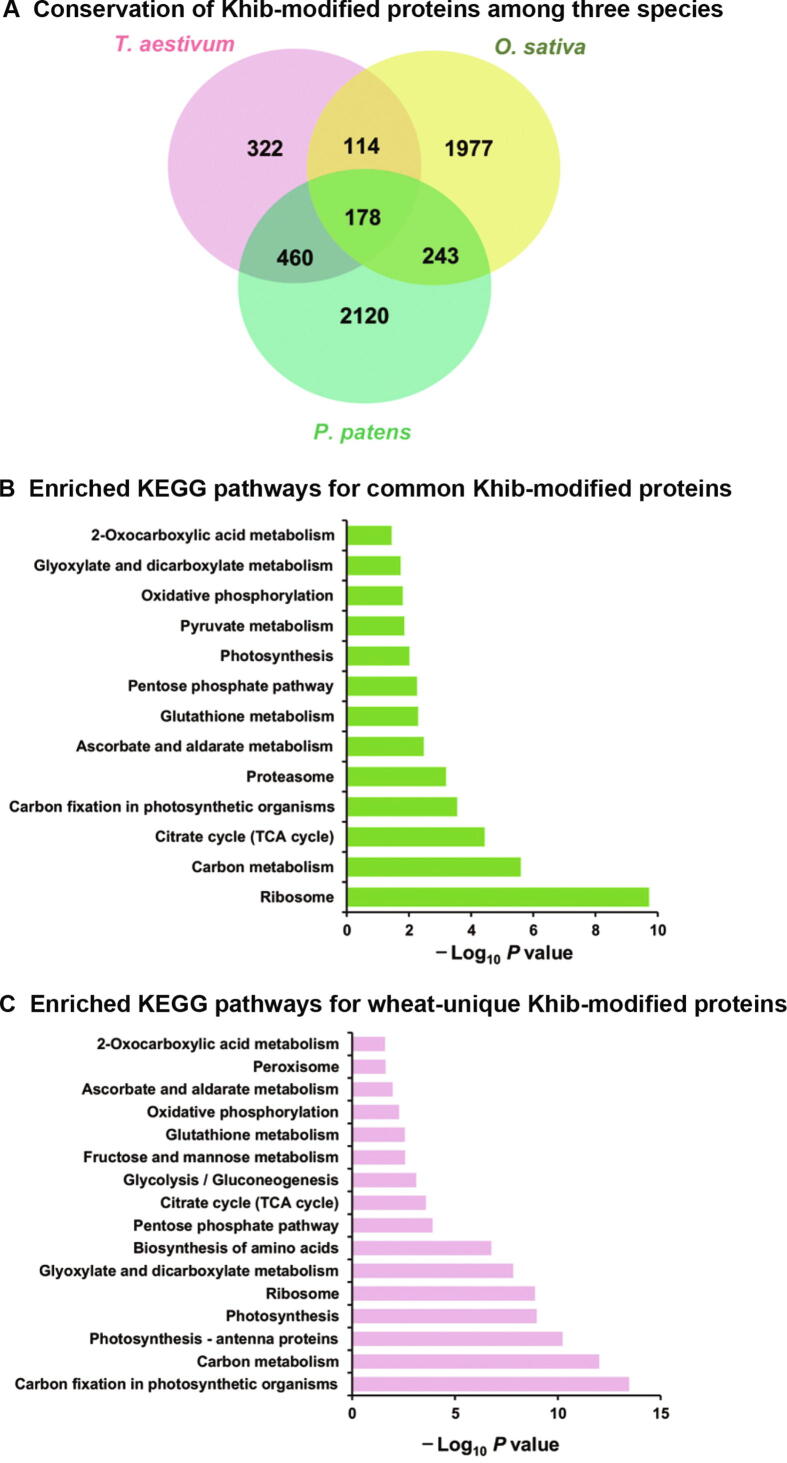
**Conservation of Khib-modified proteins in plants and uniqueness in wheat** **A.** All identified Khib-modified proteins in wheat (*T. aestivum*) compared with *O. sativa* and *P. patens*. **B.** KEGG enrichment of the common Khib-modified proteins identified in wheat, *O. sativa*, and *P. patens*. **C.** KEGG enrichment of the wheat-unique Khib-modified proteins. *T. aestivum*, *Triticum aestivum* L.; *O. sativa*, *Oryza sativa*; *P. patens*, *Physcomitrella patens*.

To reveal the conservation and uniqueness of Khib among rice (*Oryza sativa*) [23], *P. patens* [41], and wheat, we utilized a BLAST search to estimate the degree of conservation and uniqueness of the Khib-modified proteins [42]. As shown in Figure 5A and Table S10, 752 (70%) of the Khib-modified proteins were orthologous proteins between wheat and the other two species. A total of 178 Khib-modified proteins were found in all three species, and KEGG enrichment analysis indicated that these orthologous proteins were abundantly enriched in ribosomes, carbon metabolism functions, citrate cycle (TCA cycle), carbon fixation in photosynthetic organisms, and proteasomes (Figure 5B; Table S11), suggesting the vital role and conservation of Khib in these different plant species. Among the 752 identified Khib-modified proteins in wheat, 460 and 114 had conserved orthologs in *P. patens* and rice, with an averaged similarity of 66.3% and 76.6%, respectively (Figure 5A; Table S10). Moreover, 322 Khib-modified proteins were unique in wheat and mainly enriched in proteins associated with carbon fixation in photosynthetic organisms, carbon metabolism, photosynthesis-antenna proteins, and photosynthesis (Figure 5C; Table S11), indicating that photosynthesis and carbon metabolism are likely significantly regulated by Khib in wheat.

#### Overlap of Khib, Kma, Ksu, and Kac

**Figure 6:**
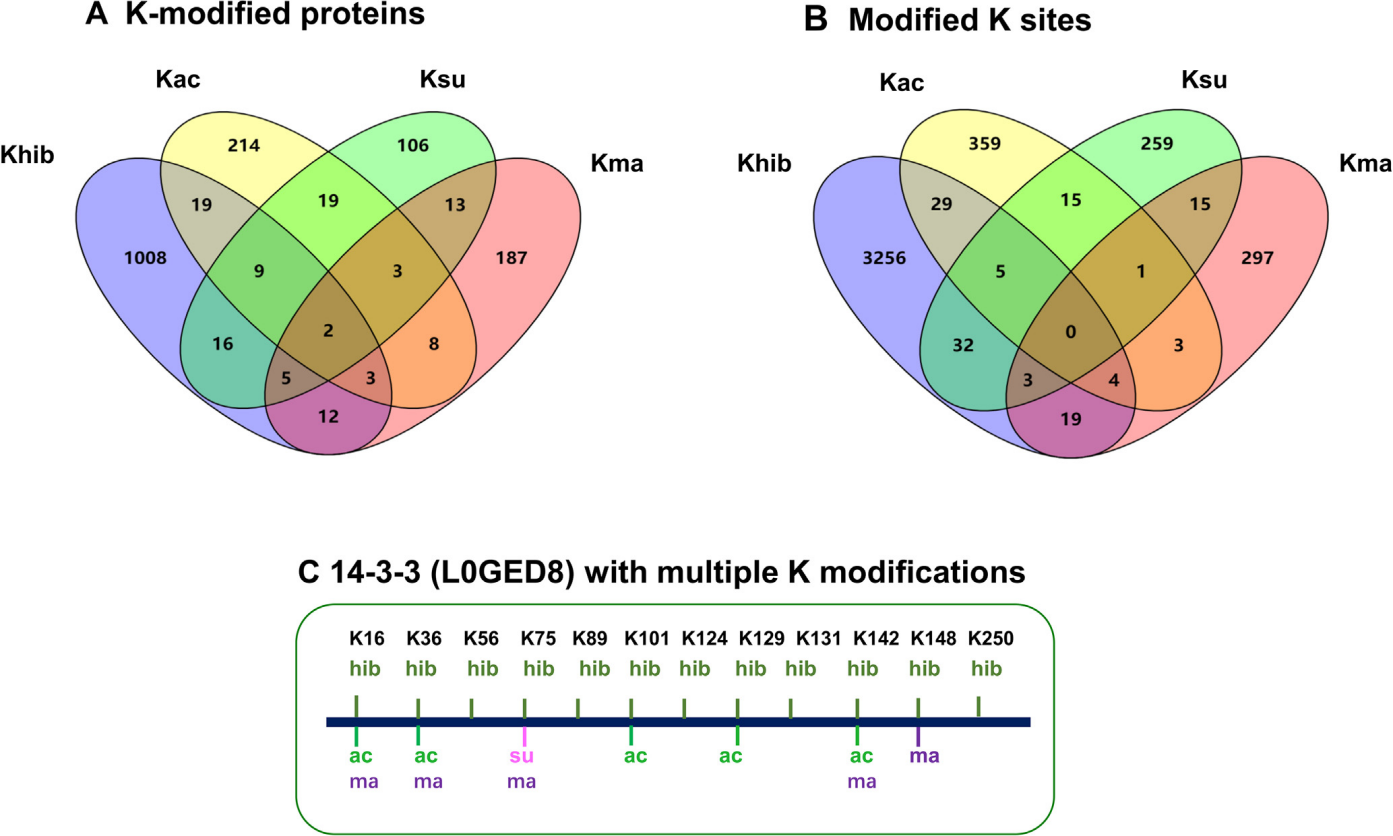
**Overlap of wheat acetylome, succinylome, malonylome, and 2-hydroxyisobutyrylome** **A.** Overlap of proteins modified by Kac, Ksu, Kma, and Khib. **B.** Overlap of K sites modified by Kac, Ksu, Kma, and Khib. **C.** A representative protein 14-3-3 (L0GED8) showing the overlap of multiple PTMs on K sites. K, lysine; Ksu, lysine succinylation; Kma, lysine malonylation; Kac, lysine acetylation.

Previous studies have indicated that PTMs play vital roles in numerous biological processes and may have the same or distinct types of modifications [35], [43], [44], [45]. To determine whether the same lysine residues were modified by Khib and other PTMs simultaneously, we compared our Khib data with published Kma, Ksu, and Kac data in common wheat [25], [26], [27]. Results revealed that 2 proteins were modified by four PTMs. Additionally, 3, 9, and 5 Khib-modified proteins were also modified by Kma and Kac, Ksu and Kac, and Ksu and Kma, respectively; 12, 16, and 19 Khib-modified proteins were also modified by Kma, Ksu, and Kac, respectively (Figure 6A; Tables S12 and S13). Moreover, 19, 32, and 29 Khib sites were found at the same positions as the Kma, Ksu, and Kac sites, respectively; 4, 3, and 5 Khib sites were found at the same positions as the Kac and Kma, Ksu and Kma, and Ksu and Kac sites, respectively (Figure 6B). Some of these Khib sites occurred in key function regions of proteins and shared the same lysine sites with other PTMs, which indicates that Khib likely possesses an important biological function.

**Supplementary Figure S5:**
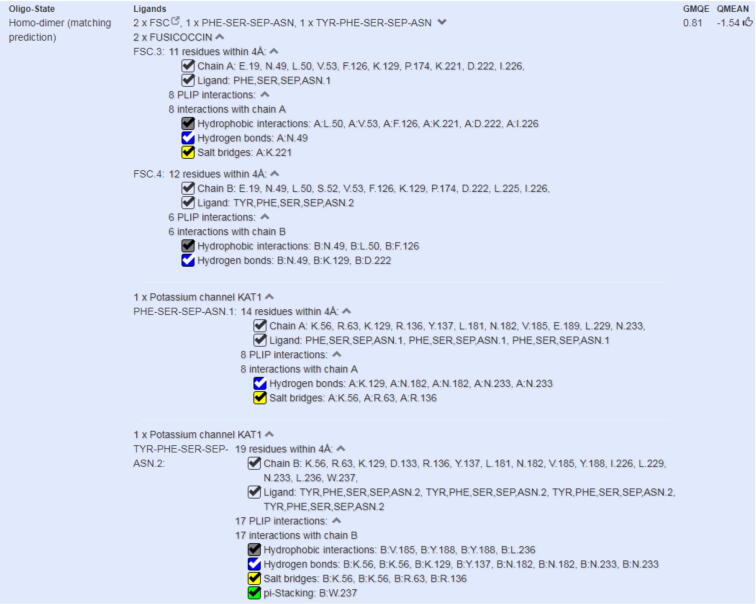
**The tertiary structure prediction information of 14-3-3 (L0GED8) using SWISS-MODEL**

For example, in protein 14-3-3 (L0GED8), ten Khib sites were determined. Among them, three Khib sites at K16, K36, and K142 were also found to be modified by Kac and Kma; one Khib site at K75 was also modified by Kma and Ksu; two Khib sites at K101 and K129 were also modified by Kac; and one Khib site at K148 was also modified by Kma (Figure 6C). The tertiary structure of 14-3-3 was predicted by SWISS-MODEL, which confirmed the importance of K56 and K129 binding to their phosphopeptide ligands (Figure S5). The Khib-modified proteins associated with the Calvin-Benson cycle, transporter, glycolysis, and ribosome were also found to be Kac-, Ksu-, or Kma-modified. These findings suggest that the same protein or lysine residue has multiple PTMs, and their combinatorial effects may regulate the function of wheat proteins.

#### Khib-modified proteins involved in photosynthesis

Khib is known to occur on nuclear, cytoplasmic, mitochondrial, and chloroplast proteins [23], [39], [40]. As a result, this PTM could be crucial for processes inside the nucleus and could be important for regulating different cellular and metabolic processes.

Proteins involved in photosynthesis are unique to plants and represent a notable proportion of the total observed Khib-modified proteins. Of all the Khib-modified proteins, 528 (49.2%) are located in the chloroplast (Figure S3A), and 37 are involved in photosynthesis (Table S8). This demonstrates that Khib may play a vital role in photosynthesis. For example, approximately 50% of the soluble leaf proteins in C3 plants is ribulose-1,5-bisphosphate carboxylase (Rubisco), which is also the most abundant protein in plant leaves. In this study, RbcL (P11383) extensively consisted of Khib sites. This protein had up to 20 independent Khib sites, such as K175, K177, K201, K252, K227, K334, and K356 (Table 1, Table S1). The IP and WB assays using a pan-anti-Khib antibody verified that RbcL was indeed Khib-modified (Figure 4A).

**Supplementary Figure S6:**
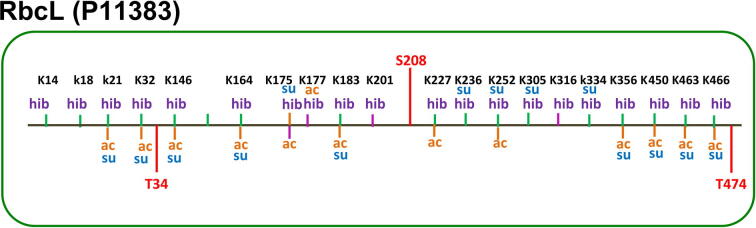
**Location of lysine, acetylation, succinylation, malonylation, 2-hydroxyisobutyrylation, and phosphorylation sites in large subunit of Rubisco (RbcL, P11383) of wheat** T34, S208, and T474 were phosphorylation sites; T, threonine; S, serine.

The majority of Khib sites occur in the key function regions [46]. For example, the RbcL ε-amino groups, K175, K177, K201, and K252, play an important role in maintaining the stability of the -CO_2_-Mg^2+^ ternary complex [47]. This implies that Khib is a key PTM in the regulation of photosynthetic CO_2_ assimilation and/or photorespiration in wheat. Moreover, the RbcL had overlapping key sites for Kac, Ksu, and Khib (Figure S6). This indicates that Kac, Ksu, and Khib possibly compete or cooperate with each other in the regulation of the same protein. A study using *Arabidopsis* indicated that RbcL was a heavily acetylated protein [46], [47]. A previous study showed that de-acetylation of RbcL could increase the maximum enzyme catalytic activity by 40%, and this may further alter the photosynthetic rate [46]. Therefore, we speculated that the activity of RbcL could be simultaneously regulated by multiple PTMs. Moreover, three Khib sites (*i.e.*, K32, K201, and K466) were found to be close to three phosphorylation sites (*i.e.*, T34, S208, and T474) [48] (Figure S6). As the phosphorylation is able to turn the corresponding protein functions on and off [39], we proposed that the interplay between Khib and nearby phosphorylation sites may result in the fine-tuning of protein functions. Other photosynthesis-related Khib-modified proteins, such as the small subunits of PS I and PS II, other subunit types of ATP synthase, and components of the photosynthetic electron transport chain, were also found to be modified by Kac or Ksu in wheat [26], [27] (Tables S12 and S13). Thus, these PTMs may be highly dynamic and play a role in the regulation of photosynthesis. Future studies should examine the effects of Khib on photosynthesis, as well as decipher how Khib coordinates phosphorylation, Kac, and Ksu in the regulation of Rubisco activity.

### Khib regulates protein functions

#### Mutation of K206 changed PGK function

In this study, many Khib-modified metabolic enzymes were identified (Table S1), suggesting that Khib may play a vital role in metabolic functions. For example, the majority of enzymes involved in tricarboxylic acid cycle (TCA), glycolysis/gluconeogenesis, and pentose phosphate pathways were modified by Khib. Altogether, 73 enzymes were determined to be Khib-modified in these three metabolic pathways; 16 of the 73 enzymes were abundantly modified and had over ten Khib sites (Table S8). Ten enzymatic reactions are required to convert glucose into pyruvate in glycolysis [49]; strikingly, seven of the key enzymes were highly modified by Khib, including GAPDH, pyruvate kinase, PGK, alpha-enolase, fructose-bisphosphate aldolase, triosephosphate isomerase, and fructose-1,6-bisphosphatase (Table S8). Among them, PGK catalyzes 3-phosphoglycerate and ATP to form 1,3-bisphosphoglycerate and ADP, which is a reversible phosphotransfer reaction. The first ATP-yielding step of glycolysis is a PGK-catalyzed reaction that is crucial for energy generation [26]. The K206 site of PGK (W5H4V7) is critical for ATP binding and was modified by Khib. These results imply that the Khib PTM may be involved in the regulation of metabolic enzymes. Thus, Khib at this position likely affects protein function through some mechanisms. However, future studies should examine the exact roles of Khib and its effects on proteins and other processes.

**Figure 7:**
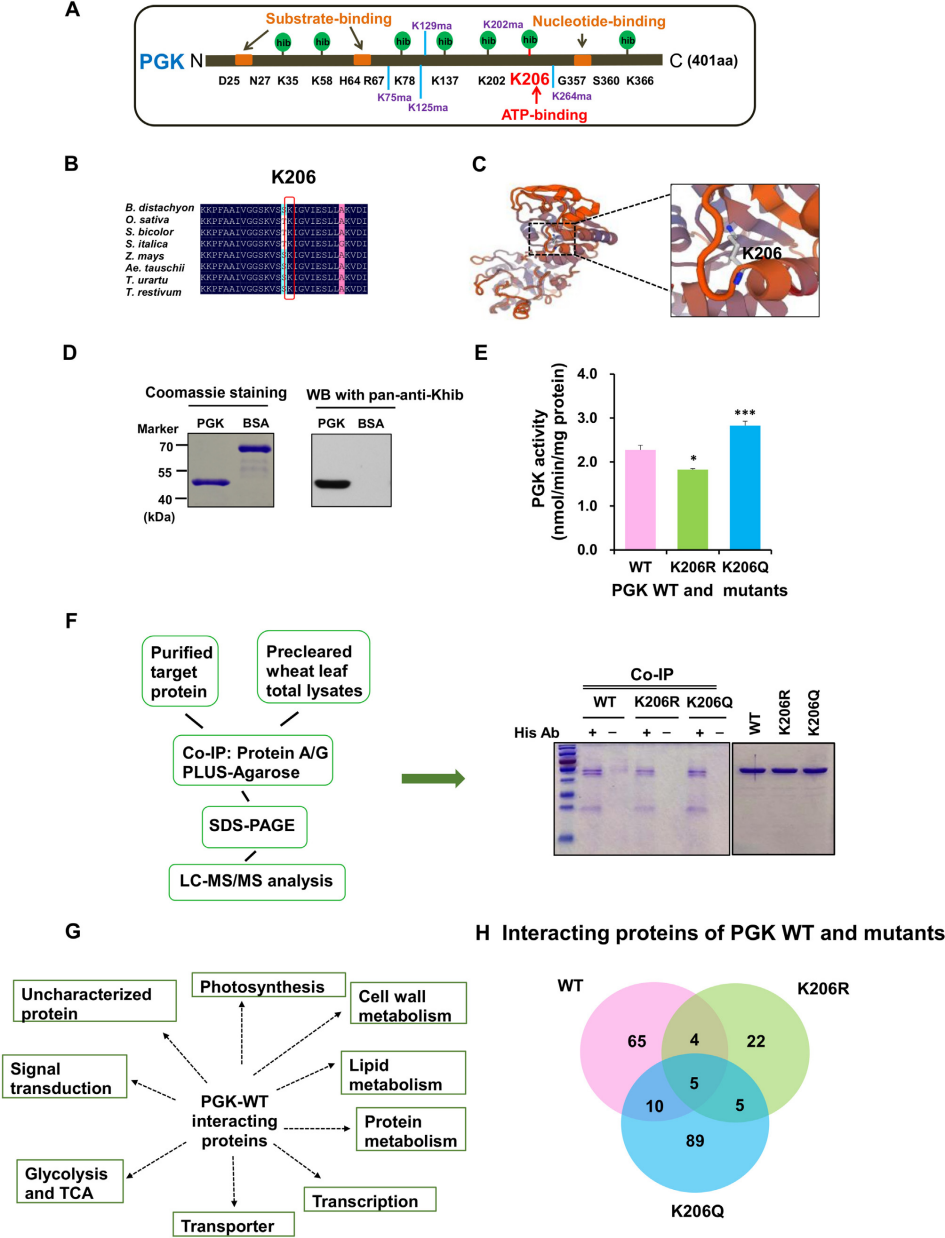
**Khib regulates enzymatic functions of wheat PGK** **A.** Location of Kma and Khib sites. **B.** Multiple sequence alignment of PGK proteins from different species. **C.** The tertiary structure prediction of PGK constructed by SWISS-MODEL. **D.** Khib levels of PGK-WT and BSA were determined by SDS-PAGE (left) and WB (right). **E.** Effect of K206 Khib on the enzymatic activity of PGK. Values are presented as the mean of three replicates. *, *P* < 0.05; ***, *P* < 0.001. **F.** The strategy for identifying PGK-interacting proteins (left); Co-IP analysis of PGK-interacting proteins (right). **G.** Proteins interacting with PGK-WT. **H.** Overlap of proteins interacting with PGK-WT, PGK-K206R, and PGK-K206Q. Five biological replicate peptides were pooled together. His Ab, anti-His-tag mouse monoclonal antibody; Co-IP, co-immunoprecipitation; WT, wild-type; Q, glutamine; R, arginine; LC-MS/MS, liquid chromatography and tandem mass spectroscopy.

In this study, three PGK proteins (*i.e.*, P12782, P12783/W5H4V7, and A0A1D6RDZ8) were determined to be Khib-modified. Among them, W5H4V7 was found to be modified by Khib at seven lysine sites and also modified by Kma at five lysine sites [25], [26], [27] (Figure 7A). These two types of PTMs occurred in key functional regions (*e.g.*, K206hib in the ATP-binding region), shared the same lysine site (K202hib/ma), or were situated near the same functional regions (*e.g.*, K35hib, K58hib, K75ma, and K366hib in the substrate-binding region), implying that PGK may be functionally regulated by Khib and Kma simultaneously. Additionally, the EMS-mutagenized wheat line with a premature stop codon in the *PGK* gene showed the delayed heading and stunted plants in comparison with wild-type (WT) controls (data not shown).

**Supplementary Figure S7:**
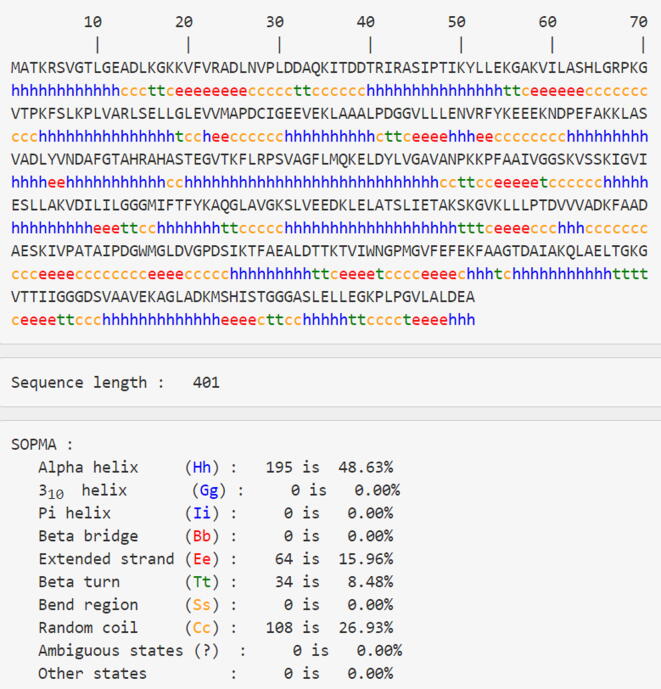
**The secondary structures analysis of phosphoglycerate kinase (PGK, W5H4V7)**

Sequence alignment showed that the K206 site was evolutionarily conserved among all eight species investigated in this study (Figure 7B), and the K206 site was modified only by Khib. The results of analyzing the protein secondary structures surrounding Khib sites revealed that four of the seven PGK Khib sites were located in α-helices, and three were located in disordered coils (Figure S7). This indicates that the enzyme has no structural preference for either ordered secondary structures or disordered structures in Khib sites. Moreover, the K206 site was located in the α-helix that is known to be directly involved in ATP binding (Figure 7C). In addition to the mutagenesis experiment, the K206 site was mutated to glutamine (K206Q, which is uncharged and could be used to simulate Khib) and arginine (K206R, which has a positive charge and cannot be modified by Khib), respectively [35]. WB indicated that the PGK was indeed Khib-modified (Figure 7D). We also assayed the enzymatic activity of the isolated proteins, which showed that K206R mutation resulted in a significant decrease in the enzymatic activity of PGK. Interestingly, we found that K206Q mutation could significantly increase the enzymatic activity as compared with the wild-type PGK (PGK-WT) (Figure 7E). A previous study indicated that PGK activity was inhibited because of Kac on K220 which disrupted the PGK binding with its ADP substrate in HEK293T cells [50]. Thus, we speculate that Khib at K206 could directly perturb the ATP-binding site.

To further examine whether the ATP-binding site mutation affects the interaction function of PGK, precleared wheat leaf proteins and PGK-WT, PGK-K206R, or PGK-K206Q were immunoprecipitated using beads **(**Figure 7F). Interactive proteins were co-immunoprecipitated with the PGK-WT protein or its two mutants and were then eluted and visualized by Coomassie blue staining. Experiments were repeated independently five times. Results revealed that the PGK-WT interacted with 84 proteins involved in multiple metabolic pathways (Figure 7G; Table S14). However, 35 proteins were found to interact with the PGK-K206R, and 109 proteins interacted with the PGK-K206Q (Figure 7H; Table S14), which is consistent with the enzymatic activity levels. Among these proteins, five were common in the PGK-WT and its two mutants (Figure 7H). These results suggest that K206 is important for PGK enzymatic activity and that Khib likely affects enzymatic activities. However, these lysine sites may also be involved with the other various PTMs existing in the organism.

#### Khib regulates 14-3-3 protein interaction

**Supplementary Figure S8:**
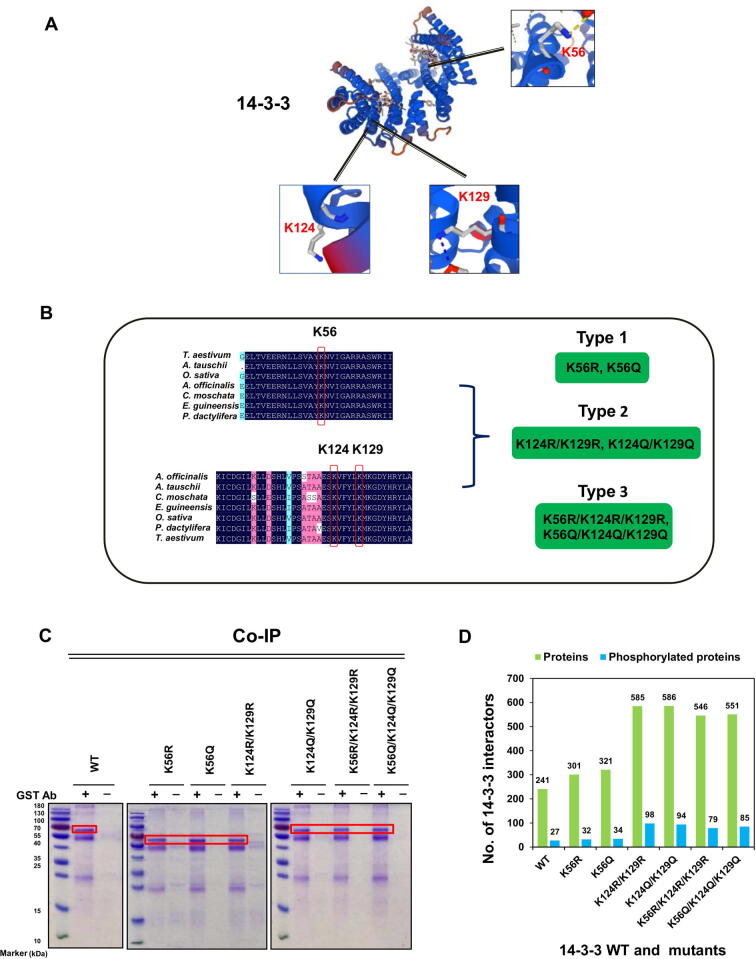
**Effects of Khib on 14-3-3 with its interactive proteins A.** The tertiary structure prediction of 14-3-3 constructed by SWISS-MODEL; the K56, K124, and K129 sites are located at an *β*-strand region. **B.** Multiple sequence alignment of 14-3-3 from different species; the conserved K56, K124, K129 site is marked by a ring. **C.** CoIP of WT, K56R, K56Q, K124R/K129R, K124Q/K129Q, K56R/K124R/K129R and K56Q/K124Q/K129Q associated proteins. GST mAb was used. **D.** LC-MS/MS analysis of 14-3-3 WT and its mutants. GST Ab, anti-GST-tag mouse monoclonal antibody.

Numerous biological processes and pathways depend on the 14-3-3 interactions to regulate key metabolic points [51]. The 14-3-3 protein could bind to specific phosphorylated motifs of target proteins by forming homodimers and heterodimers in the native state [52]. To understand the role of 14-3-3 Khib and the impact of Khib on the interactions of 14-3-3 with proteins or phosphorylated proteins in wheat, three Khib-modified lysines (K56, K124, and K129) that were highly conserved among 14-3-3 proteins were mutated (Figure S8A and B). Khib-modified lysines of 14-3-3 proteins were mutated into the R and Q forms, respectively. Interactive proteins were co-immunoprecipitated with a 14-3-3 protein or its 6 mutants before being eluted and visualized by Coomassie blue staining (Figure S8C). The analysis showed that the 14-3-3 WT interacted with 241 proteins involved in multiple metabolic pathways (Table S15). In total, 301 and 321 proteins were found to interact with 14-3-3 K56R and K56Q, respectively (Figure S8D; Table S15). More than two-fold proteins were identified to interact with double mutants (K124R/129R, n = 585; K124Q/K129Q, n = 586) or triple mutants (K56R/K124R/K129R, n = 546; K56Q/K124Q/K129Q, n = 551) when compared with WT (Table S15). Compared with WT (n = 27), more phosphorylated proteins were found to interact with the mutants, especially with double and triple mutants (K56Q, n = 34; K56R, n = 32; K124Q/K129Q, n = 94; K124R/129R, n = 98; K56Q/K124Q/K129Q, n = 85; K56R/K124R/K129R, n = 79) (Figure S8D; Table S16). The phosphopeptides identified by LC-MS/MS contained sequence-specific motifs, including RSxpSxP, RSxxpSxP, and YpT [52]. Our data indicated that phosphorylation-dependent interactions existed on the phosphopeptide-binding domain, implying that both phosphorylation and Khib may have influence on the function of 14-3-3.

### Wheat SIRTs and Zn-binding HDACs regulate Khib states

**Figure 8:**
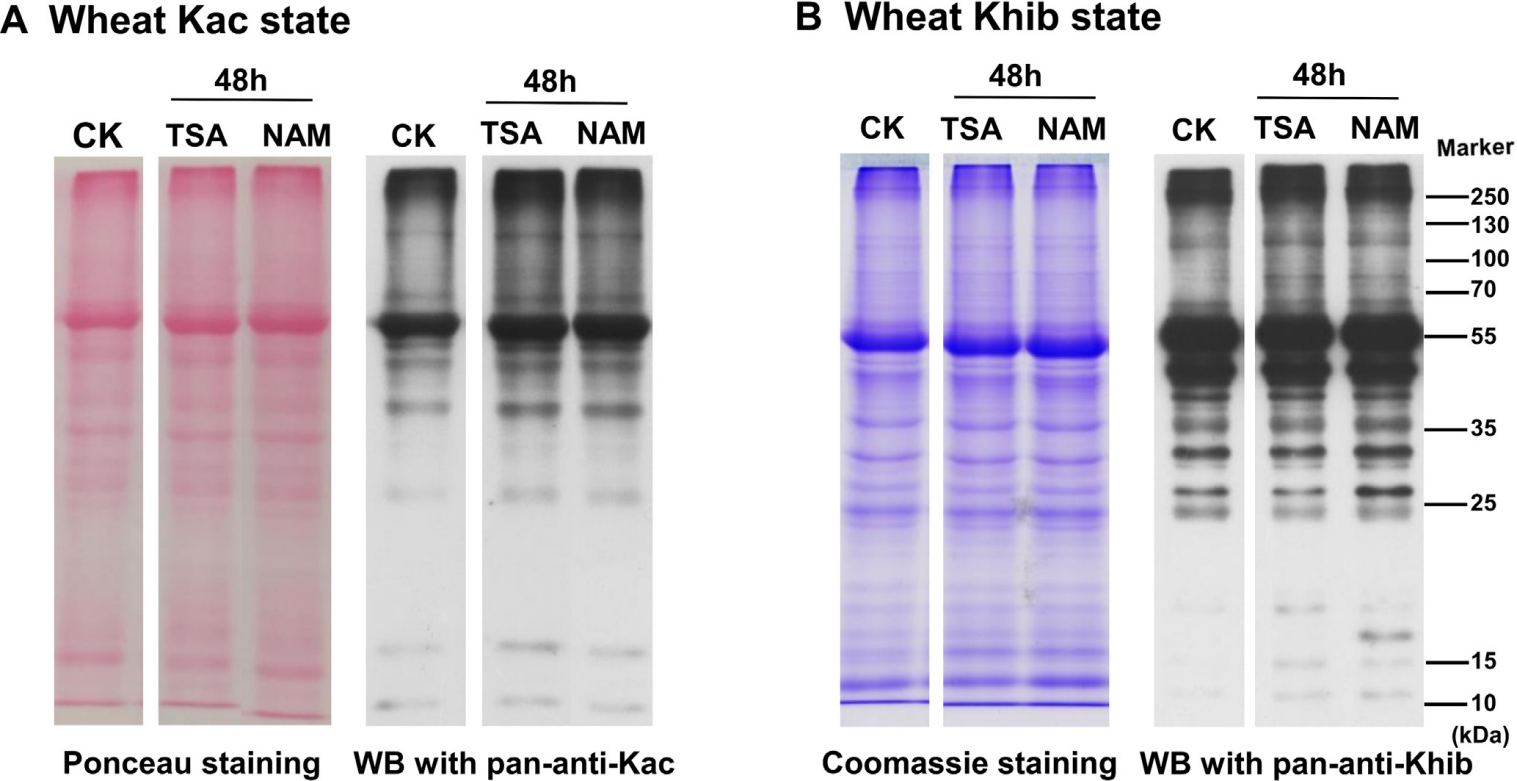
**TSA and NAM treatments change wheat Khib state** CK, control; TSA, trichostatin; NAM, nicotinamide; pan-anti-Kac antibody, pan-anti-acetyllysine antibody.

Debutyrylase and decrotonylase activities have been observed in mammalian Zn-finger HDACs [39]. For these activities, trichostatin (TSA) is an inhibitor of HDAC I and II [53], and nicotinamide (NAM) is an inhibitor for SIRT family deacetylases [54], [55]. NAM- and TSA-treated wheat seedlings were investigated to determine if wheat leaf Khib could be regulated by SIRTs or Zn-binding HDACs; this was observed by WB using the pan-anti-Khib antibody and pan-anti-Kac antibody. Compared with Kac (Figure 8A), we found that the Khib level in wheat seedlings did not increase by much after TSA and NAM treatments in the proteins with molecular weights greater than 25 kDa; however, the Khib level increased in the low-molecular-weight proteins (<25 kDa) (Figure 8B), suggesting that wheat SIRTs and Zn-binding HDACs regulate Khib. To date, the preferences and targets of the different HDACs in plants are still unknown. Compared with the genomes of *Arabidopsis* (125 Mb) [56] and rice (466 Mb) [57], common wheat possesses a larger genome (16 Gb) and is an allohexaploid (AABBDD) [58]. It has been proven that at least 18 HDACs from the three different families (RPD3/HDA1, SIRT2, and HD families) were encoded in *Arabidopsis* [59] and rice [60]. Hexaploid wheat possibly contains more HDACs, meaning that studies on the impact of HDAC regulation on Khib-modified proteins in *Triticum aestivum* L. are more difficult than in other smaller-genome species.

## Conclusion

In this study, we confirmed that Khib is an evolutionarily-conserved PTM in wheat and its progenitors. Based on affinity purification and high-resolution LC-MS/MS, a global 2-hydroxyisobutyrylome was obtained in common wheat for the first time, and a total of 3348 Khib sites on 1047 proteins were identified. Analyses showed that Khib participate in many kinds of biological processes, such as photosynthesis, glycolysis, and protein metabolism. IP and WB confirmed that Khib-modified proteins are present endogenously. Mutagenesis experiments demonstrated that Khib on the K206 site of PGK is a key regulatory modification regulating its activity and affects its interactions with substrates. Moreover, WB indicated that wheat SIRTs and Zn-binding HDACs mainly regulate Khib of the low-molecular-weight proteins. This work improves our understanding of this novel lysine PTM, and the large set of Khib-modified proteins identified in common wheat will act as an important data source for future functional studies in plants.

## Materials and methods

### Plant materials and growth conditions

Wheat progenitors, including *T. urartu* (PI428185) with an AA genome, *A. speltoides* (PI542245) with an SS genome, *A. tauschii* var. *strangulate* (AS2393) with a DD genome, tetraploid *T. durum* Langdon (AABB), and hexaploid wheat Ak58, were planted as detailed in our previous report [42]. Leaf tissues from two seedling leaf periods were collected. Afterward, samples were frozen in liquid nitrogen for subsequent protein extraction and experimentation.

### Protocol for 2-hydroxyisobutyrylome

Leaf tissues collected from two seedling leaf periods were used to extract proteins (File S1). Protein concentrations were examined according to the manufacturer’s instructions of the BCA Protein Assay Kit (Catalog No. P0012, Beyotime, Beijing, China). Trypsin digestion, HPLC fractionation, affinity enrichment, LC-MS/MS analysis, and database search are shown in File S1.

### Bioinformatics methods

UniProt-GOA database (http://www.ebi.ac.uk/GOA/) was used to carry out the GO annotation. InterProScan was used to annotate proteins’ domain functional descriptions. Protein pathways were annotated using the KEGG database. Wolfpsort was used to predict subcellular localization, and Motif-X was used to analyze the model of sequences. Genes/Proteins (STRING) database v10.5 was used for determining PPIs; the PPI network derived from STRING was visualized in Cytoscape [61].

### Co-immunoprecipitation assay

Wheat leaf (0.5 g) proteins were lysed in lysis buffer (1% Triton X-100, 20 mM Tris-HCl pH 8.0, 2 mM DTT, 800 μM PMSF, and 250 mM sucrose), supplemented with a protease inhibitor cocktail. Co-immunoprecipitation (Co-IP) was carried out by incubating with or without 25 μl of anti-RbcL (Catalog No. RGR2030, Real Time Biotech, Shanghai, China). Thirty microliter of Protein A/G PLUS-Agarose (Catalog No. sc-2003, Santa Cruz Biotech, Santa Cruz, CA) was added to the solution and incubated for 6 h on ice. After incubation, we washed the bound proteins four times with phosphate buffer solution (PBS) buffer, and then suspended them in 40 μl of PBS buffer. Proteins in PBS buffer were analyzed by sodium dodecyl sulfate-polyacrylamide gel electrophoreses (SDS-PAGE) and WB.

### Wheat germ protein expression system

Transcripts of CAT (A0A1D5YMQ8), 14-3-3 (L0GED8), GST (Q9SP56), GAPDH (A0A1D5TTT4), and PGK (W5H4V7) were amplified by PCR from the cDNA of wheat Ak58 (Table S17). After restriction digestion, the transcripts of CAT, 14-3-3, GST, PGK, and GAPDH were cloned into pFN19K HaloTag T7 SP6 Flexi Vector (Catalog No. G184A, Promega, Madison, WI) with *Sgf* I and *Pme* I restriction enzyme cutting sites; a 6×His tag was added before TAG. Each recombinant Halo-His-gene was expressed using a 30 μl TNT SP6 High-Yield Wheat Germ Protein Expression System (Catalog No. L3261, Promega). Expression levels were determined by SDS-PAGE and WB. Magne Halo Tag Beads (Catalog No. G728A, Promega) were used to purify recombinant proteins according to the manufacturer’s instructions. We used Halo TEV protease (Catalog No. L3261, Promega) to cleave the Halo tag and then capture the recombinant His-protein in elution buffer (137 mM NaCl, 2mM KH_2_PO_4_, 10 mM Na_2_HPO_4_, 2.7 mM KCl, and 0.005% IGEPALR CA-630). Lastly, we analyzed the recombinant proteins by SDS-PAGE and WB.

### Site-directed mutagenesis and prokaryotic expression

PGK (W5H4V7) was cloned into the prokaryotic expression vector, PET28a, using *Nde* I and *Xho* I as restriction enzyme cutting sites (Table S17). We performed site-directed mutagenesis of K206 (K206R, AAG-AGG; K206Q, AAA-CAA) within the PET28a plasmid using the Fast Site-Directed Mutagenesis Kit (Catalog No. KM101, Tiangen Biotech, Beijing, China). PCR was conducted using site-specific primers (Table S17). Positive mutants were verified by DNA sequencing. *E. coli* BL21 (DE3) was used to express recombinant PGK-WT, PGK-K206R, and PGK-K206Q. A His-tag Protein Purification Kit (Catalog No. P2226, Beyotime) was used to purify proteins. The collected PGK-WT recombinant proteins and two mutants were analyzed by SDS-PAGE and WB. For PGK, enzyme activity was measured by following the decrease in absorbance at 340 nm, which is due to the oxidation of NADH. The assay was conducted using commercial assay kits (Comin Biotechnology, Suzhou, China) according to the manufacturer’s instructions. Experiments were performed in triplicate.

Using *Bam*H I and *Not* I as restriction enzyme cutting sites, 14-3-3 was also cloned into the prokaryotic expression vector PGEX-6P-1 (Table S17). Site-directed mutagenesis of K56 (K56R, AAA-AGA; K56Q, AAA-CAA), K124/K129 (K124R/K129R, AAA-AGA/AAA-AGA; K124Q/K129Q, AAA-CAA/AAA-CAA), and K56/K124/K129 (K56R/K124R/K129R, AAA-AGA/AAA-AGA/AAA-AGA; K56Q/K124Q/K129Q, AAA-CAA/AAA-CAA/AAA-CAA) within the PGEX-6P-1 plasmid was performed using the Fast Site-Directed Mutagenesis Kit (Catalog No. KM101, Tiangen Biotech). PCR was conducted using site-specific primers (Table S17). Positive mutants were verified by DNA sequencing. *E*. *coli* BL21 (DE3) was used to express recombinant 14-3-3 WT, K56R, K56Q, K124R/K129R, K124Q/K129Q, K56R/K124R/K129R, and K56Q/K124Q/K129Q proteins. A GST-tag Protein Purification Kit (Catalog No. P2262, Beyotime) was used to purify proteins. We analyzed the collected 14-3-3 WT recombinant proteins and six mutants by SDS-PAGE and WB.

### Co-IP of wheat leaf proteins with PGK/14-3-3 and its mutants

Wheat leaf extracts were precleared per the following method. Approximately 1 mg of whole wheat leaf extract (in line with Co-IP) was added to 30 µl of agarose conjugate (Catalog No. sc-2003, Santa Cruz Biotech) suspended in 1 ml of PBS buffer. This solution was incubated at 4 °C for 30 min, and then centrifuged with pellet beads at 3000 r/min for 30 s at 4 °C. The supernatant was transferred to a new microcentrifuge tube and kept at 4 °C. Then, 200 µg of PGK-WT, PGK-K206R, or PGK-K206Q proteins and 25 µl of His-tag mouse monoclonal antibody (Catalog No. M30111, Abmart, Shanghai, China) were added and incubated at 4 °C overnight with mixing. After incubation, the mix was washed four times with PBS buffer, suspended in 40 μl PBS buffer, and separated by SDS-PAGE. Based on molecular weight, we cut each lane into 2 or 3 segments. Trypsin was used to digest gel segments. Peptides of PGK-WT, PGK-206R, or PGK-206Q from five biological replicates were pooled together. Pooled tryptic peptides were used for LC-MS/MS analyses. Co-IP of the wheat leaf proteins with 14-3-3 WT and its six mutants was conducted using the method detailed above.

### NAM- and TSA-treated seedlings

Wheat Ak58 seedlings from the two leaf periods were treated with 0.5 µM TSA (Catalog No. HY-15144, MedChemExpress, Monmouth Junction, NJ) or 10 mM NAM (Catalog No. HY-B0150, MedChemExpress) for 48 h. Leaf tissues from control (without treatment), TSA-treated, and NAM-treated seedlings were collected and then frozen in liquid nitrogen for subsequent protein extraction and experimentation.

### Antibodies

The antibodies, anti-RbcL (Catalog No. RGR2030, Real Time Biotech), plant Actin monoclonal (Catalog No. A01050, Abbkine, Beijing, China), anti-HaloTag monoclonal (Catalog No. G921A, Promega), anti-His-tag mouse monoclonal (Catalog No. M30111, Abmart), anti-GST-tag mouse monoclonal (Catalog No. M20007, Abmart), pan-anti-Khib (Catalog No. PTM-801, PTM Biolabs, Hangzhou, China), and pan-anti-acetyllysine (pan-anti-Kac, Catalog No. PTM-101, PTM Biolabs) were used in this study. We also used goat anti-mouse lgG HRP (Catalog No. M21001, Abmart), goat anti-rabbit lgG HRP (Catalog No. M21002, Abmart), and HRP-conjugated AffiniPure mouse anti-rabbit IgG light chain (Catalog No. AS061, ABclonal, Wuhan, China) as secondary antibodies in this study.

### Statistical analysis

A one-way analysis of variance and Duncan’s multiple range test were carried out using SPSS v17.0 statistical software.

## Data availability

The MS-based proteomic data in this study have been deposited in the ProteomeXchange Consortium via the PRIDE partner repository [62] (ProteomeXchange: PXD012666), which are publicly accessible at http://www.ebi.ac.uk/pride.

## CRediT author statement

**Ning Zhang:** Conceptualization, Methodology, Funding acquisition, Software, Investigation, Resources, Writing - original draft, Visualization, Project administration, Supervision. **Lingran Zhang:** Software, Investigation, Resources, Validation, Formal analysis. **Linjie Li:** Software, Investigation, Resources. **Junyou Geng:** Validation, Formal analysis. **Lei Zhao:** Validation, Formal analysis. **Yan Ren:** Visualization, Project administration, Supervision. **Zhongdong Dong:** Visualization, Project administration, Supervision. **Feng Chen:** Conceptualization, Methodology, Funding acquisition, Writing - review & editing, Visualization, Project administration, Supervision. All authors have read and approved the final manuscript.

## Competing interests

The authors have declared no competing interests.

## Supplementary material

Supplementary File S1 **The procedure of plant protein extract, trypsin digestion, HPLC fractionation, affinity enrichment, LC-MS/MS analyses, and database search**

Supplementary Figure S1 **Examples of raw mass spectra in common wheat**

Supplementary Table S1 **Quantitative results of lysine 2-hydroxyisobutyrylation sites identified in common wheat**

Supplementary Table S2 **Structural features analyses of 2-hydroxyisobutyrylation proteins in common wheat**

Supplementary Table S3 **Subcellular locations of 2-hydroxyisobutyrylation proteins in common wheat**

Supplementary Table S4 **GO annotation 2-hydroxyisobutyrylation proteins in common wheat**

Supplementary Table S5 **GO enrichment of 2-hydroxyisobutyrylation proteins in common wheat**

Supplementary Table S6 **KEGG enrichment of 2-hydroxyisobutyrylation proteins in common wheat**

Supplementary Table S7 **Protein domain functional description of 2-hydroxyisobutyrylation proteins in common wheat**

Supplementary Table S8 **KEGG pathways of 2-hydroxyisobutyrylation proteins in common wheat**

Supplementary Table S9 **Information of the interaction network among 2-hydroxyisobutyrylation proteins in common wheat using STRING**

Supplementary Table S10 **Overlap of the Khib-modified proteins among common wheat, rice, and *Physcomitrella patens***

Supplementary Table S11 **KEGG enrichment of common 2-hydroxyisobutyrylation proteins identified in wheat, rice, and *Physcomitrella patens*, and specific 2-hydroxyisobutyrylation proteins identified in wheat**

Supplementary Table S12 **Overlap of wheat 2-hydroxyisobutyrylation, malonylation, succinylation, and acetylation proteins**

Supplementary Table S13 **Overlap of wheat 2-hydroxyisobutyrylation, malonylation, succinylation, and acetylation sites**

Supplementary Table S14 **LC-MS/MS analyses of PGK-WT, PGK-K206R, and PGK-K206Q associated proteins**

Supplementary Table S15 **LC-MS/MS analyses of 14-3-3-WT, K56R, K56Q, K124R/K129R, K124Q/K129Q, K56R/K124R/K129R and K56Q/K124Q/K129Q associated proteins**

Supplementary Table S16 **Phosphorylation LC-MS/MS analyses of 14-3-3-WT, K56R, K56Q, K124R/K129R, K124Q/K129Q, K56R/K124R/K129R and K56Q/K124Q/K129Q associated proteins**

Supplementary Table S17 **Primer sequences used in this study**
