# Supplementary material for: Global Profiling of 2-hydroxyisobutyrylome in Common Wheat
Source: Genomics Proteomics Bioinformatics. 2021 Feb 11;20(4):688–701. doi: 10.1016/j.gpb.2020.06.008 (PMC9880814; doi:10.1016/j.gpb.2020.06.008)
Supplement: Supplementary File S1 — The procedure of plant protein extract, trypsin digestion, HPLC fractionation, affinity enrichment, LC-MS/MS analyses, and database search [file mmc1.docx]

**File S1 The procedures of plant protein extract, trypsin digestion,** **HPLC fractionation, affinity enrichment, LC-MS/MS analysis and database search**

**For plant protein extract**

Sample was first grinded by liquid nitrogen, then the powder was transferred to 5 mL centrifuge tube and sonicated three times on ice using a high intensity ultrasonic processor (Scientz, Zhejiang, China) in lysis buffer (including 1% TritonX-100, 10 mM dithiothreitol, 1% Protease Inhibitor Cocktail, 50 μM PR-619, 3 μM TSA, 50 mM NAM, and 2 mM EDTA). An equal volume of Tris-saturated phenol (pH 8.0) was added; then, the mixture was further vortexed for 5 min. After centrifugation (4 °C, 10 min, 5 000 g), the upper phenol phase was transferred to a new centrifuge tube. Proteins were precipitated by adding at least four volumes of ammonium sulfate-saturated methanol and incubated at −20 °C for at least 6 h. After centrifugation at 4 °C for 10 min, the supernatant was discarded. The remaining precipitate was washed with ice-cold methanol once, followed by ice-cold acetone for three times. The protein was redissolved in 8 M urea and the protein concentration was determined with BCA kit according to the manufacturer’s instructions.

**Trypsin digestion**

For digestion, the protein solution was reduced with 5 mM dithiothreitol for 30 min at 56 °C and alkylated with 11 mM iodoacetamide for 15 min at room temperature in darkness. The protein sample was then diluted by adding 100 mM NH_4_HCO_3_ to urea with concentration less than 2M. Finally, trypsin was added at 1:50 trypsin-to-protein mass ratio for the first digestion overnight and 1:100 trypsin-to-protein mass ratio for a second 4 h-digestion.

**HPLC fractionation**

The tryptic peptides were fractionated into fractions by high pH reverse-phase HPLC using Thermo Betasil C18 column (5 μm particles, 10 mm i.d., 250 mm length). Briefly, peptides were first separated with a gradient of 8% to 32% acetonitrile (pH 9.0) over 60 min into 60 fractions. Then, the peptides were combined into fractions and dried by vacuum centrifuging.

**Affinity enrichment**

To enrich Khib modified peptides, tryptic peptides dissolved in NETN buffer (100 mM NaCl, 1 mM EDTA, 50 mM Tris-HCl, 0.5% NP-40, pH 8.0) were incubated with pre-washed antibody beads (Lot number PTM-804, PTM Bio, Hangzhou, China) at 4 °C overnight with gentle shaking. Then the beads were washed four times with NETN buffer and twice with H_2_O. The bound peptides were eluted from the beads with 0.1% trifluoroacetic acid. Finally, the eluted fractions were combined and vacuum-dried. For LC-MS/MS analyses, the resulting peptides were desalted with C18 ZipTips (Millipore, Massachusetts, USA) according to the manufacturer’s instructions.

**LC-MS/MS analysis**

The tryptic peptides were dissolved in 0.1% formic acid (solvent A), directly loaded onto a home-made reversed-phase analytical column (15 cm length, 75 μm i.d.). The gradient was comprised of an increase from 6% to 23% solvent B (0.1% formic acid in 98% acetonitrile) over 26 min, 23% to 35% in 8 min and climbing to 80% in 3 min then holding at 80% for the last 3 min, all at a constant flow rate of 400 nl/min on an EASY-nLC 1000 UPLC system.

The peptides were subjected to NSI source followed by MS/MS in Q Exactive^TM^ Plus (Thermo, Massachusetts, USA) coupled online to the UPLC. The electrospray voltage applied was 2.0 kV. The m/z scan range was 350 to 1800 for full scan, and intact peptides were detected in the Orbitrap at a resolution of 70,000. Peptides were then selected for MS/MS using NCE setting as 28 and the fragments were detected in the Orbitrap at a resolution of 17,500. A data-dependent procedure that alternated between one MS scan followed by 20 MS/MS scans with 15.0 s dynamic exclusion. Automatic gain control was set at 5E4.

**Database search**

The resulting MS/MS data were processed using Maxquant search engine (v.1.5.2.8). Tandem mass spectra were searched against UniProt_*Triticum* database (116722 sequences, released at 2018-04) concatenated with reverse decoy database. Trypsin/P was specified as cleavage enzyme allowing up to 4 missing cleavages. The mass tolerance for precursor ions was set as 20 ppm in First search and 5 ppm in Main search, and the mass tolerance for fragment ions was set as 0.02 Da. Carbamidomethyl on Cys was specified as fixed modification and lysine 2-hydroxyisobutyrylation, acetylation on protein N-terminal and oxidation on Met were specified as variable modifications. FDR was adjusted to < 1% and minimum score for modified peptides was set > 40.
