# Supplementary material for: Global Profiling of 2-hydroxyisobutyrylome in Common Wheat
Source: Genomics Proteomics Bioinformatics. 2021 Feb 11;20(4):688–701. doi: 10.1016/j.gpb.2020.06.008 (PMC9880814; doi:10.1016/j.gpb.2020.06.008)

Serine hydroxymethyltransferase (A0A0C4BJE5)

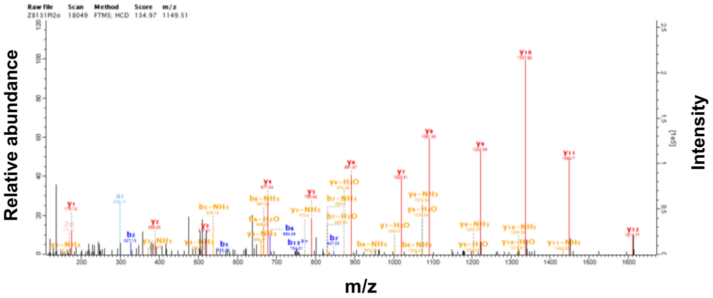

ATP synthase subunit gamma (A0A1D5RZX1)

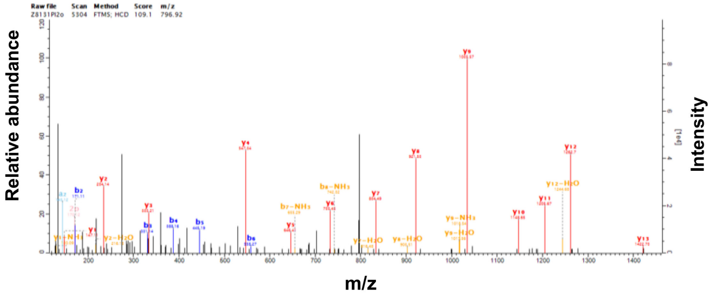

Protein TIC110, chloroplastic (A0A1D5SSJ7)

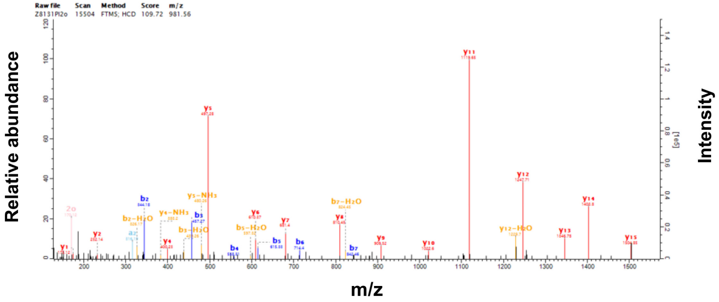

Heat shock protein 90 (F4Y593)

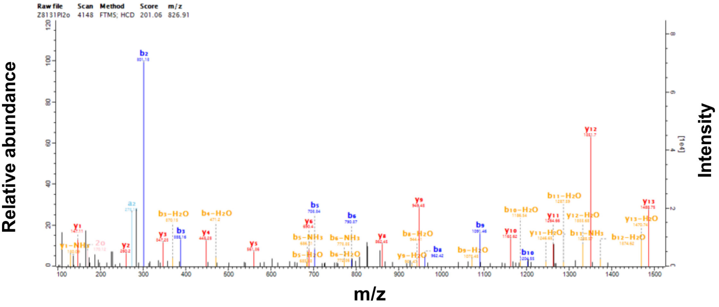

Cytochrome f (P05151)

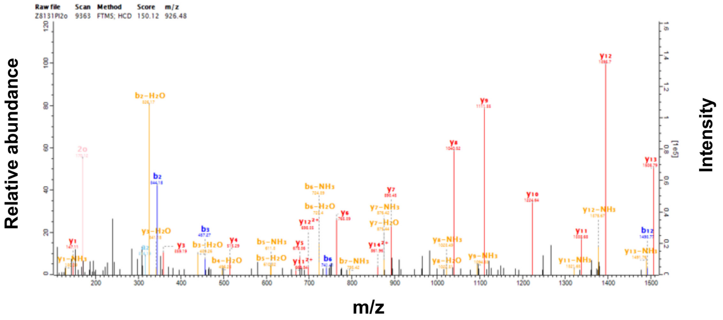

Glutathione S-transferase (Q9SP56)

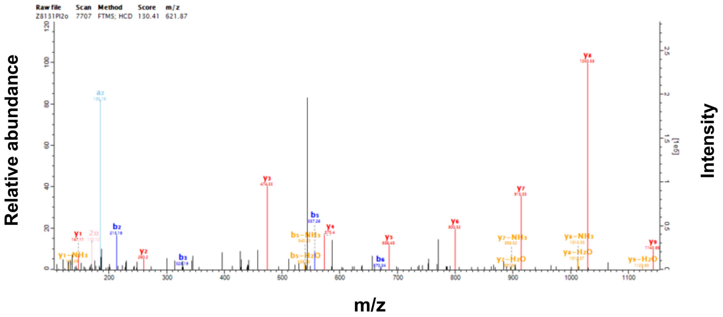

NADPH-protochlorophyllide oxidoreductase (W4ZSC8)

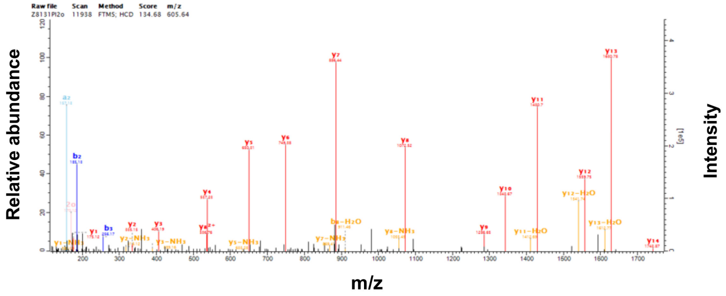

Oxygen-evolving enhancer protein 3-1, chloroplastic (W5C4P1)

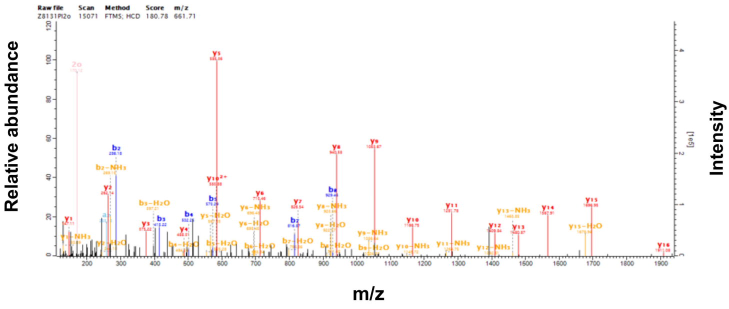

Probable mediator of RNA polymerase II transcription subunit 37e (W5FEE5)

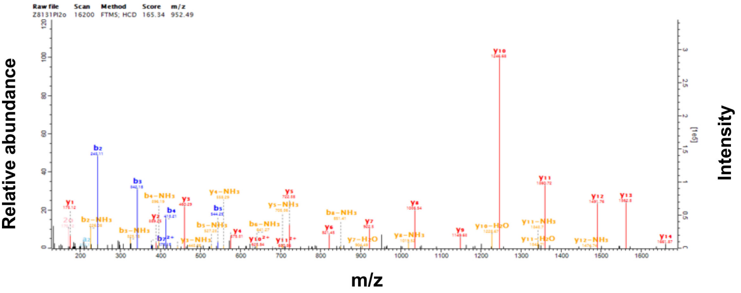

50S ribosomal protein L9, chloroplastic (W5GIE6)

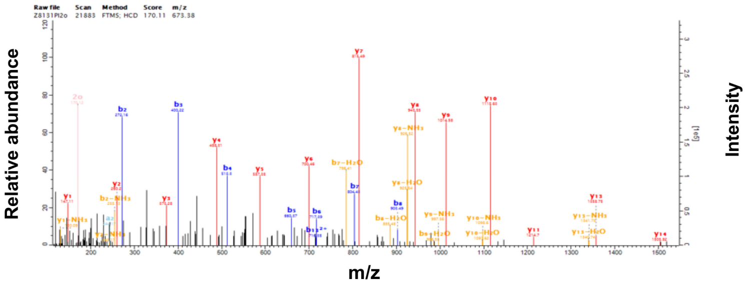

Supplement: Supplementary Figure S1 — Examples of raw mass spectra in common wheat [file mmc2.pdf]
